# The Clinical Development of Disopyramide

**Published:** 1976

**Authors:** S. I. Ankier

**Affiliations:** Roussel Laboratories


### DISOPYRAMIDE — SCIENTIFIC AND CLINICAL SEMINAR

On Monday 11th October, 1976 a discussion group was held under the Chairmanship of Dr. H. G. Mather at Blagdon on the anti-arrhthymic agent disopyramide (Rythmodan, Roussel). The material presented here summarises the two papers given.

### The Clinical Development of Disopyramide

**Dr. S. I. Ankier,**
Roussel Laboratories

Disopyramide was synthesised around 1959. Since its structure is not related to any other known anti-arrhythmis drug, one can only suppose that its discovery was serendipity. Initial studies on dysopyramide led workers to conclude that disopyramide was very similar in its activity to quinidine but we now know this is not completely true. Unfortunately; the original French clinical investigations were subjective and anecdotal in nature. Perhaps medicine is more of a science than an art in the UK, while in France it tends to be more of an art than a science. Certainly, the French have a reputation for impressionistic art. As a consequence several studies were set up in the UK, Canada, South Africa, Australia, Germany and Scandinavia between 1973 and 1975 to re-examine the pharmacology of disopyramide and establish its clinical usefulness in carefully controlled trials. I was lucky enough to initiate and co-ordinate much of that particular experience, which was stimulating, fascinating and rewarding. Most of these comprehensive investigations led to an International Symposium on Disopyramide at the Royal College of Physicians in 1975.

Pharmacologically, disopyramide has anti-cholinergic activity which is about 1/200 to 1/1200 of that of atropine and this action explains the side-effects which are most often seen clinically when high doses are used or if a patient has a significant degree of renal impairment. Disopyramide is less despressant than quinidine, propanolol or verapamil on myocardial metabolism, and its haemodynamic effects are relatively small. Disopyramide is a local anaesthetic with a potency similar to that of lignocaine. It has no alpha or beta adrenoreceptor blocking activity, is not a neuro-muscular or ganglion blocker, and has no anti-histaminic, diuretic, anorectic or overt CNS activity in animals. In animals, disopyramide is anti-arrhythmic against both atrial and ventricular arrythmias. The drug was usually more active than quinidine or procainamide.

In man, slow intravenous injection of disopyramide causes a slight transient negative inotropic effect. Cardiac output is either unaffected or slightly and transiently reduced, there is either no effect on the heart rate or a slight transient increase, but the blood pressure is usually maintained or slightly and transiently increased. These minimal effects are accompanied by an increase in peripheral resistance. Disopyramide causes either no effect on conduction time in the atrium or a slight increase, while the conduction time through the His-Purkinje system and ventricle is usually increased. The conduction time through the AV node is usually unaffected by disopyramide. Animal studies have shown that phases 0 and 4 depolarisation are depressed and in man the effective refractory period is increased in both the atrium and the ventricle. There is a slight increase in the effective refractory period of the AV node.

In patients with the Wolff-Parkinson-White syndrome, the conduction time and the effective refractory period in anomalous pathways are increased, and even blocked, both in the antigrade and in the retrograde direction, making disopyramide useful in the treatment of the WPW syndrome.

Disopyramide is very effective against ventricular premature beats, atrial and ventricular tachycardia, as well as maintaining sinus rhythm following electroconversion of atrial fibrillation. There is no absolute incompatibility betwen disopyramide and any other anti-arrythmic agent, but it should be used with caution in combination with other negatively inotropic drugs. Disopyramide is contra-indicated in the presence of complete heart block and patients with severe heart failurea should be digitalised prior to disopyramide therapy. To-date extensive clinical use has shown that disopyramide is strikingly free of serious side-effects.

In summary, experience shows that disopyramide is an effective and relatively safe anti-arryhthmic agent, especially against ventricular arrhythmias.

**References**

1. The Journal of International Medical Research, 1976, 4, Supplement (1).
2. Proceedings of the Disopyramide (Rythmodan) Seminar (1977) — Viking Press Ltd., London.

